# Radiological indicators to predict the application of assistant intubation techniques for patients undergoing cervical surgery

**DOI:** 10.1186/s12871-020-01153-0

**Published:** 2020-09-17

**Authors:** Bingchuan Liu, Yanan Song, Kaixi Liu, Fang Zhou, Hongquan Ji, Yun Tian, Yong Zheng Han

**Affiliations:** 1 Department of Orthopaedics, Peking University Third Hospital, Beijing, China; 2 Beijing Key Laboratory of Spinal Disease Research, Beijing, China; 3 Department of Anesthesiology, Peking University Third Hospital, 49 North Garden Rd, Haidian District, Beijing, 100191 China

**Keywords:** Difficult laryngoscope, Assistant intubation technique, Radiological indicator, Clinical study

## Abstract

**Background:**

We aimed to distinguish the preoperative radiological indicators to predict the application of assistant techniques during intubation for patients undergoing selective cervical surgery.

**Methods:**

A total of 104 patients were enrolled in this study. According to whether intubation was successfully accomplished by simple Macintosh laryngoscopy, patients were divided into Macintosh laryngoscopy group (*n* = 78) and Assistant technique group (*n* = 26). We measured patients’ radiographical data via their preoperative X-ray and MRI images, and compared the differences between two groups. Binary logistic regression model was applied to distinguish the meaningful predictors. Receiver operating characteristic (ROC) curve and area under the curve (AUC) were used to describe the discrimination ability of indicators. The highest Youden’s index corresponded to an optimal cut-off value.

**Results:**

Ten variables exhibited significant statistical differences between two groups (*P* <  0.05). Based on logistic regression model, four further showed correlation with the application of assistant techniques, namely, perpendicular distance from hard palate to tip of upper incisor (X2), atlanto-occipital gap (X9), angle between a line passing through posterior-superior point of hard palate and the lowest point of the occipital bone and a line passing through the anterior-inferior point and the posterior-inferior point of the second cervical vertebral body (Angle E), and distance from skin to hyoid bone (MRI 7). Angle E owned the largest AUC (0.929), and its optimal cut-off value was 19.9° (sensitivity = 88.5%, specificity = 91.0%). the optimal cut-off value, sensitivity and specificity of other three variables were X2 (30.1 mm, 76.9, 76.9%), MRI7 (16.3 mm, 69.2, 87.2%), and X9 (7.3 mm, 73.1, 56.4%).

**Conclusions:**

Four radiological variables possessed potential ability to predict the application of assistant intubation techniques. Anaesthesiologists are recommended to apply assistant techniques more positively once encountering the mentioned cut-off values.

## Background

Airway management is regarded as the most important aspect in clinical anesthesia and a successful intubation remains crucial for surgical procedures [1]. Clinically, there are many factors associated with difficulty of intubation during laryngoscopy, including head-neck trauma [2], airway abnormalities [3], gastroesophageal reflux disease [4], hard to open mouth [5], impaired cervical mobility [6], etc. The incidence of difficulty to undergo laryngoscopy and intubation ranges widely among different studies, and patients with cervical spondylosis have a higher incidence of difficult laryngoscopy (17.1%) [7] than those without cervical spondylosis (7.3%) [8]. This might in turn cause unexpected difficult airways in large proportion of patients, significantly increasing the morbidity and mortality rates [9]. Difficulty during laryngoscopy due to unexpected situations brings huge challenge to anesthesiologists. Under such circumstances, multiple attempts of intubation and application of assistant intubation techniques are considered inevitable.

A pre-planned induction strategy involves consideration of various interventions that are designed to facilitate intubation during difficult airway conditions. The interventions that are intended to manage difficult airway include, but are not limited to [10]: (1) video-assisted laryngoscopy, (2) lighted stylets (e.g., shikani, light wand), (3) fiberoptic-guided intubation, (4) supraglottic airway for ventilation and intubation (e.g., intubating laryngeal mask airway), and (5) invasive airway access (e.g., tracheostomy). However, the clinical application and promotion of these assisted techniques were still associated with some limitations. Firstly, overuse without any specific indications not only causes wastage of medical resources, but reduces the productivity of anesthesiologist. Secondly, the opportunity of optimal intubation might be missed when these techniques are forced to be applied under unexpected situations and multiple attempts of intubation might cause iatrogenic guttural injury. Therefore, before undergoing intubation, an effective predictive strategy that can provide anesthesiologists with information on the necessity and possibility of assistant techniques is required and considered crucial in clinical practice.

Many researchers have attempted to predict difficult of intubation by preoperative radiologic data, but very few studies have specifically targeted at the necessity and possibility of the application of assistant techniques for intubation. Hence, in the present study, this prediction was carried out based on radiological indicators of patients who underwent cervical surgery. Our study findings could provide valuable information for airway management practice, especially for patients with special conditions where traditional assessment methods such as thyromental distance, mouth opening, cervical mobility and Mallampati classification are difficult to be obtained.

## Methods

### Study design

From June 2019 to December 2019, patients who underwent elective cervical spine surgery under general anesthesia were recruited in this cohort study. Inclusion criteria of patients were as follows: (1) age ranging from 20 to 70 years; (2) with good mental health; and (3) complete radiographic and clinical materials. Exclusion criteria were as follows: patients (1) with airway tumor or foreign body; (2) severe cervical trauma; (3) cervical spine instability; (4) poor physical conditions (ASA IV or V); and (5) anticipated difficult mask ventilation. The clinical and radiological data of patients were acquired by reviewing their medical history and measuring the values on the Picture Archiving and Communication Systems (PACS). This study was approved by Medical Ethics Committee of Peking University Third Hospital, Peking University Health Science Center, Beijing (IRB00006761–2015021). Informed consents were obtained from the patients.

### Measurement of radiological indicators

**Fig. 1:**
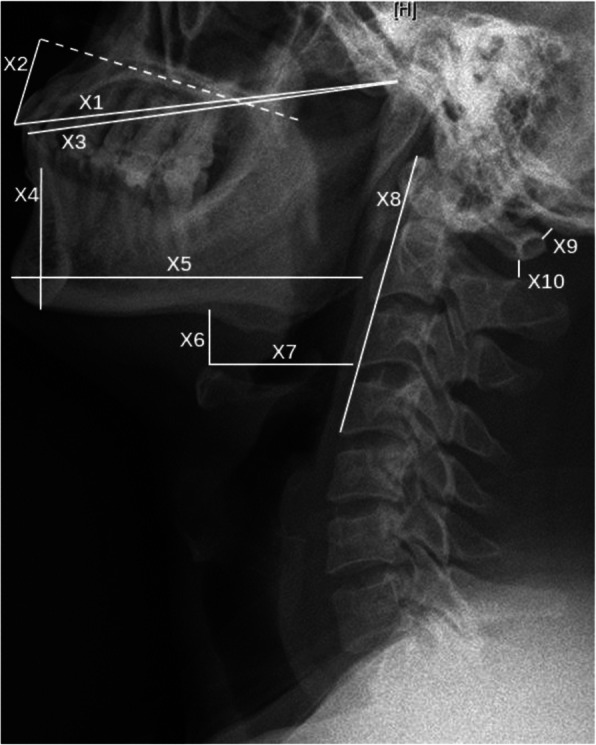
Distance indicators on the lateral cervical X-ray in the neutral position. X1: distance between temporomandibular joint and the tip of upper incisor; X2: perpendicular distance from hard palate to the tip of upper incisor; X3: distance between temporomandibular joint and the tip of lower incisor; X4: anterior depth of mandible; X5: length of mandibular body; X6: vertical distance from the highest point of hyoid bone to mandibular body; X7: horizontal distance from the highest point of hyoid bone to the border of the nearest cervical vertebra; X8: distance from the anterior-inferior border of the fourth cervical vertebra to the anterior-superior border of the first vertebra; X9: atlanto-occipital gap; X10: distance between the spinous processes of the first and second cervical vertebra

**Fig. 2:**
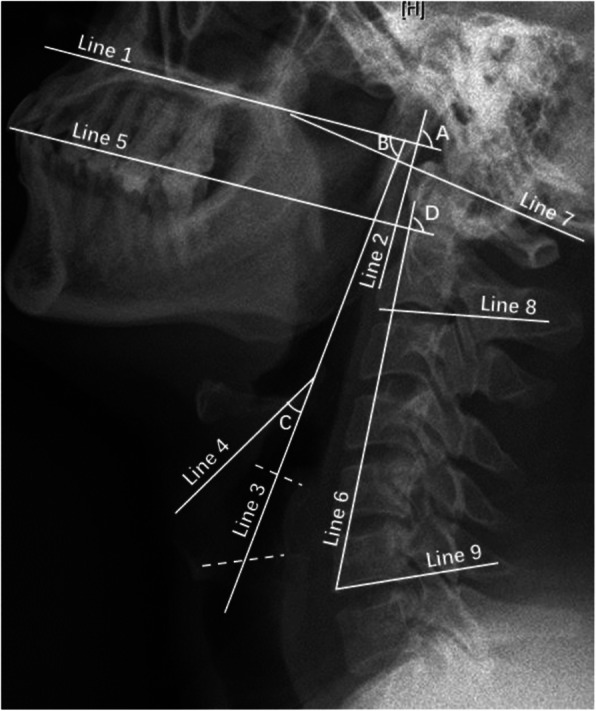
Angle indicators on the lateral cervical X-ray in the neutral position. Angle A: angle between Line 1 and Line 2; Angle B: angle between Line 1 and Line 3; Angle C: angle between Line 3 and Line 4; Angle D: angle between Line 5 and Line 6; Angle E: angle between Line 7 and Line 8; Angle F: angle between Line 8 and Line 9. (Line 1: a line parallel to hard palate; Line 2: a line passing through the anterior point of the bodies of atlas and axis; Line 3: a line passing through the airway midpoint crossing the cricoid cartilage; Line 4: a line parallel to epiglottis; Line 5: a line along the occlusal surface of maxillary teeth; Line 6: a line passing through anterior-inferior border of the sixth cervical vertebra and the most anterior aspect of the first cervical vertebra; Line 7: a line passing through the posterior-superior point of hard palate and the lowest point of the occipital bone; Line 8: a line passing through the anterior-inferior point and the posterior-inferior point of the second cervical vertebral body; Line 9: a line passing through the anterior-inferior point and the posterior-inferior point of the sixth vertebral body)

**Fig. 3:**
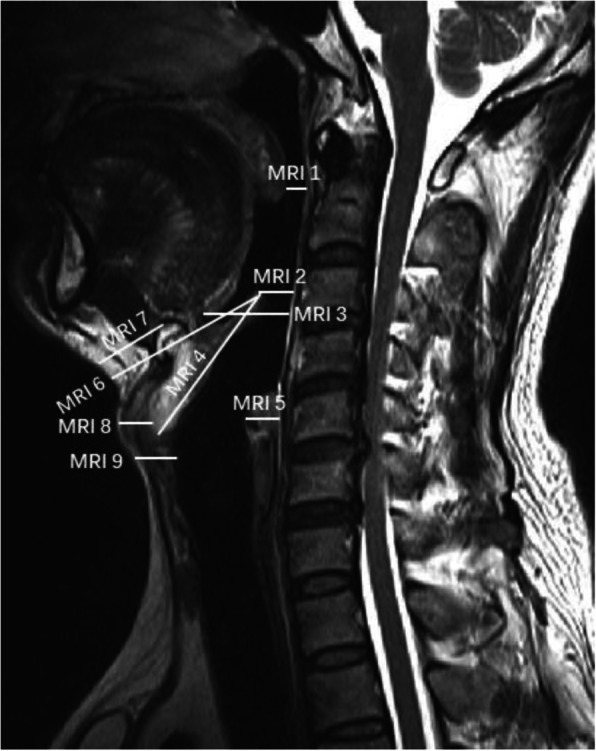
Distance indicators on the lateral sagittal neck MRI in the neutral position. MRI 1: distance between uvula and the posterior pharyngeal wall; MRI 2: distance between the tip of epiglottis and the posterior pharyngeal wall; MRI 3: distance between the base of tongue and the posterior pharyngeal wall; MRI 4: the length of epiglottis; MRI 5: distance between vocal cord and the posterior pharyngeal wall; MRI 6: distance from skin to the tip of epiglottis; MRI 7: distance from skin to hyoid bone; MRI 8: distance from skin to thyroid cartilage at the level of vocal cord; MRI 9: distance from skin to vocal cord

Radiological data were obtained by cervical X-ray examination and neck MRI (MR750; GE Medical Systems, Milwaukee, WI, USA). X-ray examination was performed by informing patients to maintain standing position and MRI scan was completed in the supine position. All X-ray and MRI data were evaluated using radiography information system (Centricity RIS-IC CE V3.0; GE Healthcare, Little Chalfont, UK) of the Peking University Third Hospital. All distance and angle indicators on cervical lateral X-rays were measured in the neutral position (Figs. 1 and 2), which indicated that the cervical spine was maintained its natural curvature without flexion and extension, and sagittal T2-weighted neck MRI indicators were also measured in neutral position (Fig. 3). All imaging indicator measurements were completed by the same orthopedic surgeon for all patients in batches. The detailed measurement methods of all parameters are described in the figure legends. Bias was avoided as orthopedic surgeon was blinded to group allocation, and not involved in the intubation and anesthesia management.

### Intubation procedure

Routine preoperative monitoring of non-invasive blood pressure, heart rate, pulse oximetry, and electrocardiography were performed. Anesthesia was induced with sufentanil (0.3 μg/kg) and propofol (2 mg/kg). For patients who lost consciousness, neuromuscular blockade was injected by rocuronium (0.6 mg/kg). The Macintosh laryngoscope was applied by senior anesthesiologists who were not involved in the preoperative radiologic assessment. Patients who were successfully intubated with Macintosh laryngoscope were assigned to the Macintosh group, and those unsuccessfully intubated with Macintosh laryngoscope were settled according to the Difficult Airway Society 2015 guidelines [11].

### Selection of different assistant intubation techniques

Patients who were unsuccessfully intubated with Macintosh laryngoscope were further dealt with assistant techniques in this study. In this study, unsuccessful intubation was defined as the clinical situation in which a conventionally trained anesthesiologist (working more than 5 years) could not successfully complete tracheal intubation less than three attempts with Macintosh laryngoscope. The video-assisted laryngoscopy is the first choice as it is easy to handle, and can be inserted into the patient’s mouth. If failed, shikani or fiberoptic-guided intubation was considered as alternative techniques. If the patient has poor oxygenation, then the intubating laryngeal mask airway should be chosen. In emergency situation or those in need for invasive airway access, tracheostomy could also be considered.

### Patient and public involvement

No patients were involved in the radiological data measurements nor were they involved in developing plans for design and accomplishment of the present study. No patients were asked to advise on interpretation. The final results will be disseminated to investigators and patients through this publication.

### Statistical analysis

SPSS 22.0 software was used to execute statistical analysis. Kolmogorov-Smirnov test assists in determining whether the distribution was normal. Continuous variables that were normally distributed were analyzed by independent-samples T test, while non-normal variables were assessed by Mann-Whitney test. Categorical data were analyzed by Chi-square test. After that, binary logistic regression model was applied to distinguish multivariate predictors. Odds ratio (OR) and 95% confidence interval (95% CI) signifies the strength of association. The receiver operating characteristic (ROC) curve was used to describe the discrimination ability of the predictive indicators. Area under the curve (AUC) was used as a quantitative index. Youden’s index (= sensitivity + specificity - 1) was calculated and the highest score was considered as an optimal predictive cut-off value. *P* <  0.05 was considered to be statistically significant.

## Results

A total of 104 patients were enrolled in this study. Based on whether assistant techniques were applied during intubation, patients were divided into Macintosh laryngoscopy group (78 cases) and Assistant technique group (26 cases). In the Assistant technique group, 4 patients underwent intubation with video-assisted laryngoscope successfully, 4 patients underwent intubation with shikani optical stylet, 15 patients with fiberoptic bronchoscopy, 2 patients with laryngeal mask airway, and 1 patient with tracheostomy tube. The average age of the patients was 51.77 years, and included 92 males and 12 females. Patients’ demographics and measurement data were displayed in Table 1. There were no significant differences in the aspects of gender, age, height, weight and BMI between two groups (*P* > 0.05). With regard to radiological measurement, ten indicators that showed significant differences between the two groups were found, namely, X2 (*P* <  0.001), X6 (*P* = 0.028), X7 (*P* = 0.001), X9 (*P* = 0.039), Angle B (*P* = 0.037), Angle E (*P* <  0.001), Angle F (*P* = 0.017), MRI 1 (*P* = 0.002), MRI 4 (*P* = 0.013), and MRI 7 (*P* <  0.001).

**Table 1:** Demographics and measurement data between two groups

Items	Macintosh laryngoscopy group (*n* = 78)	Assistant technique group (*n* = 26)	Statistic (χ^2^/z/t)	*P*-value
Male (%)	71 (91.0)	21 (80.8)	2.010	0.156
Age (years)	51.2 ± 8.6	53.4 ± 10.4	1.062	0.291
Height (cm)	170.7 ± 5.9	168.3 ± 6.6	−1.349	0.177
Weight (kg)	71.2 ± 8.2	68.2 ± 9.1	−1.524	0.128
BMI (kg/m^2^)	24.4 ± 2.7	24.0 ± 2.7	− 0.641	0.534
X1 (mm)	106.0 ± 7.7	108.0 ± 6.8	1.178	0.242
X2 (mm)	28.1 ± 3.5	33.0 ± 4.1	− 4.857	**< 0.001**
X3 (mm)	103.5 ± 7.4	104.1 ± 6.7	− 0.184	0.854
X4 (mm)	40.9 ± 3.8	39.2 ± 5.0	−1.793	0.076
X5 (mm)	98.8 ± 6.9	101.1 ± 7.9	1.396	0.166
X6 (mm)	19.3 ± 6.3	16.3 ± 4.9	− 2.225	**0.028**
X7 (mm)	44.7 ± 6.1	40.0 ± 6.2	−3.323	**0.001**
X8 (mm)	95.0 ± 7.3	93.6 ± 8.2	−0.825	0.411
X9 (mm)	7.9 ± 2.8	6.6 ± 1.7	−2.064	**0.039**
X10 (mm)	6.1 ± 1.8	6.1 ± 2.6	− 0.833	0.405
Angle A (°)	97.1 ± 7.9	95.8 ± 5.9	− 0.748	0.456
Angle B (°)	83.1 ± 7.4	80.2 ± 9.9	− 2.091	**0.037**
Angle C (°)	28.7 ± 3.9	29.4 ± 2.8	− 0.214	0.831
Angle D (°)	99.8 ± 7.7	101.6 ± 9.6	0.155	0.338
Angle E (°)	27.2 ± 6.3	14.8 ± 5.7	−8.866	**< 0.001**
Angle F (°)	15.9 ± 6.1	12.5 ± 6.3	−2.391	**0.017**
MRI 1 (mm)	8.1 ± 1.7	6.9 ± 1.8	−3.125	**0.002**
MRI 2 (mm)	7.4 ± 1.9	7.2 ± 2.4	−0.721	0.471
MRI 3 (mm)	16.7 ± 3.8	15.9 ± 3.7	−0.998	0.321
MRI 4 (mm)	39.5 ± 4.5	42.1 ± 4.2	2.541	**0.013**
MRI 5 (mm)	9.3 ± 1.7	8.7 ± 1.3	−1.653	0.101
MRI 6 (mm)	46.5 ± 5.94	44.8 ± 6.1	−1.263	0.209
MRI 7 (mm)	20.7 ± 4.2	16.1 ± 6.1	−4.212	**< 0.001**
MRI 8 (mm)	11.6 ± 3.6	11.3 ± 6.2	−1.723	0.085
MRI 9 (mm)	8.6 ± 2.1	9.8 ± 4.3	−1.156	0.248

Based on the binary logistic regression model (Forward: LR) presented in Table 2, among the ten mentioned indicators with significant differences between the two groups, 4 indicators showed further correlation with the application of assistant techniques during intubation. These included X2 (*P* = 0.005, OR = 0.526), X9 (*P* = 0.019, OR = 3.175), Angle E (*P* < 0.003, OR = 1.723), and MRI 7 (*P* = 0.018, OR = 1.375), and their 95% CI were (0.337 to 0.819), (1.213 to 8.309), (1.206 to 2.463), (1.058 to 1.796), respectively.

**Table 2:** The binary logistic regression model (Forward: LR) of the enrolled variables

Items	B	SE	*P*-value	OR	95% CI
X2	−0.643	0.226	0.005	0.526	0.337, 0.819
X9	1.155	0.491	0.019	3.175	1.213, 8.309
Angle E	0.544	0.182	0.003	1.723	1.206, 2.463
MRI 7	0.321	0.135	0.018	1.378	1.058, 1.796

**Fig. 4:**
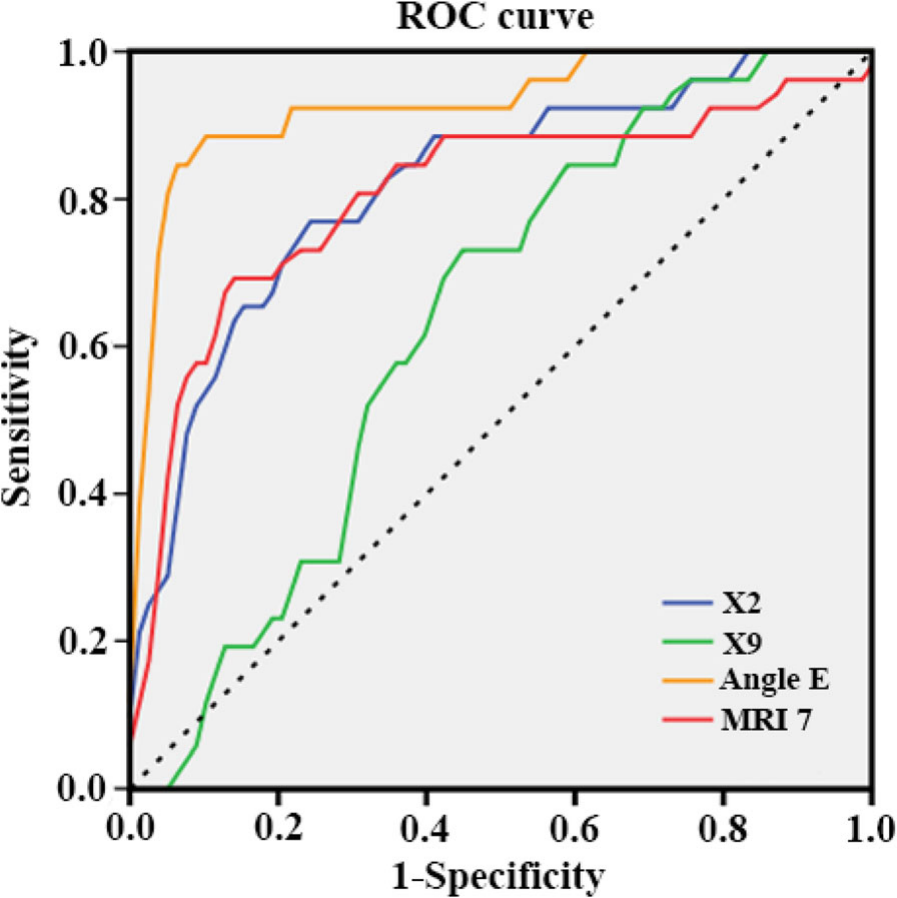
ROC curve of the four indicators including X2, X9, Angle E, MRI 7. (X2: perpendicular distance from hard palate to the tip of upper incisor; X9: atlanto-occipital gap; Angle E: angle between Line 7 and Line 8; MRI 7: distance from skin to hyoid bone. Line 7: a line passing through the posterior-superior point of hard palate and the lowest point of the occipital bone; Line 8: a line passing through the anterior-inferior point and the posterior-inferior point of the second cervical vertebral body)

The ROC curve and the AUC were used to understand the predictive ability of the 4 radiological predictors established by the logistic regression model. As shown by Table 3 and Fig. 4, Angle E owned the largest AUC (0.929) (95% CI was 0.873 to 0.986), and the AUCs of X2 and MRI 7 were higher than 0.8. According to the highest Youden’s index, the optimal cut-off value of Angle E was 19.9° (sensitivity = 88.5%, specificity = 91.0%), and the optimal cut-off value, sensitivity and specificity of other variables were X2 (30.1 mm, 76.9, 76.9%), MRI7 (16.3 mm, 69.2, 87.2%), and X9 (7.3 mm, 73.1, 56.4%).

**Table 3:** The AUC and the optimal cut-off value based on the highest Youden’s index

Items	AUC	Highest Youden’s index	Optimal cut-off value	Sensitivity	Specificity
Angle E	0.929	0.795	19.9	0.885	0.910
X2	0.819	0.538	30.1	0.769	0.769
MRI 7	0.805	0.564	16.3	0.692	0.872
X9	0.636	0.295	7.3	0.731	0.564

## Discussion

Due to the existence of cervical degeneration, instability or spondylosis, difficult laryngoscopy has a higher incidence in patients undergoing elective cervical spine surgery. To reduce the unnecessary attempts and increase the efficiency and accuracy of the anaesthesiologist during application of assistant techniques during intubation, and based on patients’ preoperative cervical X-ray and MRI images, this study was conducted to distinguish radiological predictors that were reported in the previous literature in difficult conditions where assisted techniques should be prepared and applied in a more positive manner and where necessary. To our knowledge, only few studies have focused on the prediction of the application of assisted techniques according to the preoperative radiological measurements. Our findings would hold great value in providing references to clinical anesthesia.

Angle E, which is the angle between a line passing through the posterior-superior point of hard palate and the lowest point of the occipital bone and a line passing through the anterior-inferior point and the posterior-inferior point of the second cervical vertebral body, could reflect the upper cervical spine mobility. Less Angle E seen in our study implied the limited flexion of upper cervical spine, which might result in difficult laryngoscopy. The occipitoatlantal junction contributes 23.0°–24.5° of flexion/extension of the skull and the atlantoaxial joint provides an additional 10.1°–22.4° [12] movement. Hence, the upper cervical spine contributes a vast majority of flexion and extension of the cervical spine mobility. Besides, the atlantoaxial joint serves as an important hub structure that connects the skull and spine, where many important vessels and nerves were located [13]. The limitation of flexion and extension might increase the risk of disastrous iatrogenic injuries if laryngoscopy is forced to be applied [14]. In our study, Angle E demonstrated the best ability to predict the necessity of assistant technique application (AUC = 0.929), and the cut-off value of 19.9° had the highest sensitivity (88.5%) and specificity (91.0%). The results suggested that if the Angle E was less than 19.9° in clinical intubation, higher vigilance and more positive application of assistant techniques are required.

X2, the perpendicular distance from hard palate to the tip of upper incisor, was also an effective radiological indicator in predicting the difficult laryngoscopy in this study (AUC = 0.819). The patients in Assistant technique group were detected with longer X2 distance (33.0 ± 4.1 mm vs. 28.1 ± 3.5 mm, *P* < 0.001). In a sense, longer X2 distance caused by bucktooth and abnormal hard palate indicates shorter inter-incisor gap and smaller oral cavity space anatomically [15, 16]. These two limiting factors further restrict the intraoral operation of laryngoscopy and create difficulty in exposing the glottis [17, 18]. In this study, the optimal cut-off value of X2 was 30.1 mm (sensitivity = 76.9%, specificity = 76.9%), and this indicated that a X2 distance of more than 30.1 mm reminded us the application of assisted techniques during intubation more possibly and necessarily.

MRI 7 is the distance from skin to hyoid bone. Adhikari et al. [19] have reported that this distance could be used to distinguish difficult and easy laryngoscopies, and found that it was higher in patients in difficult laryngoscopy group when compared with easy laryngoscopy group (1.69 cm, 95% CI 1.19 to 2.19 vs 1.37 cm, 95% CI 1.27 to 1.46) in a study of 51 American patients who underwent intubation in neutral position without a pillow. Besides, MRI 7 also had higher specificity and sensitivity for predicting difficult airway management, and this is because the hyoid acts as a vital factor of the upper airway, which was connected to tongue by genioglossus muscle and to the larynx via the hyoepiglottic and thyrohyoid [20]. In our study, the shorter distance from skin to hyoid bone indicated difficult laryngoscopy, which was different from the result of Adhikari [19]. The reason for this might be that the shorter MRI 7 could suggest connection of anatomical structures to the hyoid bone that were located more anteriorly and lower, which might in turn influence the exposure of glottis during laryngoscope examination. In our study, the distance from skin to hyoid bone acts as a moderate accuracy predictor with an AUC = 0.805. It was significantly different between the Macintosh laryngoscope and Assistant technique groups (20.7 ± 4.2 mm vs 16.1 ± 6.1 mm, *P* < 0.001). This result was consistent with some previous studies [21, 22], in which the distance from skin to hyoid bone as measured by ultrasound had certain predictive function for difficult laryngoscopy.

X9 is the atlanto-occipital gap, and the distance between the occipital bone and first cervical vertebra in patients undergoing intubation in neutral position. Patients with atlantooccipital distance impairment had a higher prevalence of difficulty laryngoscopy [23, 24]. X9 is related to occipito-atlanto complex, and is associated with mandibular protrusion. Patients with lesions in the occipito-atlanto complex had a higher prevalence of difficult airway than those with disease below the complex [23]. Besides, shorter X9 distance might reflect decreased motion range and slight fusion of atlanto-occipital joint to some extent. In our study, atlanto-occipital gap was significantly different between Macintosh laryngoscopy group and Assistant technique group (7.9 ± 2.8 mm vs. 6.6 ± 1.7 mm, *P* = 0.039). However, the AUC of X9 was 0.636, representing low accuracy and its optimal cut-off value was 7.3 mm with a sensitivity of 73.1% and specificity of 56.4%.

However, there are some limitations in our study. Firstly, the present study included a relatively small number of patients, and larger sample size and a multi-center study might make the results more convincing. Secondly, this was a retrospective study, and a prospective study for predicting the necessity and possibility of assistant technique application during intubation might have the potential to provide more references to clinical anesthesia. Additionally, some measurement errors might exist because the measurements were completed by a single surgeon.

## Conclusions

In summary, four radiological parameters were recognized to predict the application of assistant intubation techniques in this study. Based on the optimal cut-off values of each preoperative predictor, the possibility of difficult airway is warned, and the anaesthesiologist should then apply the assistant technique more positively before many attempts during intubation.

## Data Availability

The datasets generated and/or analyzed during the present study will be available from the corresponding author on reasonable request.

## Abbreviations

ASA: American Society of Anesthesiologists
PACS: picture archiving and communication systems
MRI: magnetic resonance imaging
ROC: receiver operating characteristic
AUC: area under the curve
